# Comparative Insights into COVID-19 and Tuberculosis: Clinical Manifestations, Inflammatory Markers, and Outcomes in Pulmonary Versus Extrapulmonary Tuberculosis and *SARS-CoV-2* Co-Infection

**DOI:** 10.3390/jcm14082782

**Published:** 2025-04-17

**Authors:** Camil Mihuta, Adriana Socaci, Patricia Hogea, Emanuela Tudorache, Monica Simina Mihuta, Cristian Oancea

**Affiliations:** 1 Department of Doctoral Studies, “Victor Babes” University of Medicine and Pharmacy, 300041 Timisoara, Romania; camil.mihuta@umft.ro; 2 Clinical Hospital for Infectious Diseases and Pneumology “Dr. Victor Babes”, 300041 Timisoara, Romania; hogea.patricia@umft.ro (P.H.); emanuela.tudorache@umft.ro (E.T.); oancea@umft.ro (C.O.); 3 Center for Research and Innovation in Precision Medicine of Respiratory Diseases (CRIPMRD), “Victor Babes” University of Medicine and Pharmacy, 300041 Timisoara, Romania; 4 Department of Biology and Life Sciences, Faculty of Medicine, “Vasile Goldis” Western University of Arad, 310025 Arad, Romania; 5 Department of Pulmonology, “Victor Babes” University of Medicine and Pharmacy, 300041 Timisoara, Romania; 6 Department of Pediatrics, Faculty of Medicine, “Victor Babes” University of Medicine and Pharmacy, 300041 Timisoara, Romania; simina.mihuta@umft.ro; 7 Center of Molecular Research in Nephrology and Vascular Disease, Faculty of Medicine, “Victor Babes” University of Medicine and Pharmacy, 300041 Timisoara, Romania

**Keywords:** COVID-19, extrapulmonary tuberculosis, pulmonary tuberculosis, *SARS-CoV-2*

## Abstract

**Background**: Tuberculosis and COVID-19 co-infection poses significant clinical challenges, with pulmonary TB (PTB) and extrapulmonary TB (extraPTB) potentially influencing disease progression and outcomes differently. This study aims to compare the clinical manifestations, inflammatory markers, and outcomes between PTB and extraPTB patients with *SARS-CoV-2* co-infection. **Methods**: A retrospective, cross-sectional study was conducted on 55 hospitalized adults with TB-COVID-19 co-infection from March 2020 to March 2022. Patients were divided into PTB (n = 32) and extraPTB (n = 23) groups. Demographic, clinical, laboratory, and imaging data were collected and analyzed using statistical models, including ANCOVA, LASSO regression, and Random Forest classification, to identify key predictors of hospitalization duration and mortality. **Results**: PTB patients had significantly lower BMI, worse oxygenation status, and greater lung involvement on CT compared to extraPTB patients. CRP was elevated in PTB, while IL-6 levels were higher in extraPTB. Hospitalization duration was primarily influenced by inflammatory and coagulation markers (IL-6, D-dimer, neutrophil count, systemic inflammatory index), while higher BMI was associated with shorter stays. Mortality risk was strongly correlated with oxygenation impairment (worst SpO_2_, SpO_2_ at diagnosis), inflammatory burden (CRP, LDH), and CT severity score, rather than TB localization. **Conclusions**: TB localization did not independently affect hospitalization duration or mortality risk. Instead, severe lung involvement, systemic inflammation, and hypoxemia were the strongest predictors of poor outcomes. These findings emphasize the importance of early risk stratification based on respiratory and inflammatory markers to optimize patient management. Further research is needed to clarify the long-term impact of TB-COVID-19 co-infection, particularly in extraPTB cases.

## 1. Introduction

Tuberculosis (TB) remains a major global health concern, with approximately a quarter of the population harboring latent *Mycobacterium tuberculosis* (MTB) infection [1,2]. While most cases are pulmonary (PTB), around 25% present as extrapulmonary TB (extraPTB), affecting various organs and posing diagnostic challenges due to its diverse manifestations and the need for invasive or advanced imaging techniques [3,4,5,6,7].

The COVID-19 pandemic has significantly impacted TB control efforts, disrupting screening, diagnosis, and treatment programs [8]. Since 2019, *SARS-CoV-2* has caused widespread morbidity and mortality, with over 774 million cases and 7 million deaths reported by early 2024 [9]. Beyond its respiratory effects, COVID-19 affects multiple organ systems and has strained healthcare resources, leading to increased TB-related mortality for the first time in over a decade, as reported by the WHO [8,10].

Beyond these structural challenges, the interaction between TB and COVID-19 presents a complex pathophysiological interplay. Both diseases have the potential to exacerbate each other’s progression, as TB is known to compromise immune function, rendering individuals more vulnerable to severe *SARS-CoV-2* infection [11,12]. COVID-19 can induce profound immune dysregulation, which may either trigger the reactivation of latent TB or accelerate the progression of active TB disease [13]. The inflammatory response associated with *SARS-CoV-2*, particularly the cytokine storm characterized by elevated levels of interleukin-6 (IL-6), tumor necrosis factor alpha, and C-reactive protein (CRP), may disrupt granuloma formation, a key immunological mechanism that controls MTB infection [14,15]. Furthermore, immunosuppressive treatments commonly used for severe COVID-19, such as corticosteroids and biologic agents, may inadvertently increase the risk of TB reactivation or progression, further complicating clinical management [16].

Both PTB and COVID-19 exhibit similarities in airborne transmission, primary respiratory involvement, overlapping symptoms, and shared social determinants, such as poverty and overcrowding, which contribute to their widespread prevalence. Despite these parallels, their underlying pathophysiological mechanisms are distinctly different. Unlike PTB, which primarily compromises lung function and shares similar respiratory symptoms with COVID-19, extraPTB involves diverse organ systems, resulting in variable clinical presentations. ExtraPTB cases often lack the hallmark features of PTB, such as cough, hemoptysis, and night sweats, instead presenting with symptoms related to the specific organ involved, such as lymphadenopathy, joint pain, abdominal discomfort, or neurological deficits. These differences raise critical questions regarding the susceptibility of extraPTB patients to severe COVID-19 outcomes, the potential impact of co-infection on organ-specific immune responses, and whether extraPTB alters the course of COVID-19 compared to PTB [4,6].

Given these uncertainties, there is a pressing need for targeted research to better understand the clinical and paraclinical dynamics of TB/COVID-19 co-infection, particularly in differentiating the impact of *SARS-CoV-2* infection on PTB versus extraPTB. Our previous work has focused on analyzing differences between *SARS-CoV-2* infection alone versus *SARS-CoV-2*-PTB and extraPTB co-infection, respectively [17,18]. Building on our prior work, this study aims to provide a comparative analysis of *SARS-CoV-2* infection in patients with PTB versus those with extraPTB. By systematically assessing differences in clinical presentation, biomarkers, imagistic findings, and disease outcomes, we seek to elucidate whether one type of TB confers a different risk profile for severe COVID-19 or alters the inflammatory and immune response compared to the other. This comparative approach will enhance our understanding of the complex interplay between TB and COVID-19 and may contribute to refining diagnostic algorithms, optimizing treatment strategies, and improving clinical management for co-infected patients.

## 2. Materials and Methods

### 2.1. Study Design

This retrospective, cross-sectional, randomized study included 55 adult patients, aged 19–91 years, who were hospitalized and managed at the Victor Babeș Hospital of Infectious Diseases and Pneumoftiziology in Timișoara. Data collection was conducted between March 2020 and March 2022, during the COVID-19 pandemic.

This study compared two distinct groups of patients:

- Group 1: Patients diagnosed with pulmonary tuberculosis (PTB) and *SARS-CoV-2* coinfection (n = 32).
- Group 2: Patients diagnosed with extrapulmonary tuberculosis (extraPTB) and *SARS-CoV-2* coinfection (n = 23). ExtraPTB cases included pleural TB (n = 13), tuberculous lymphadenitis (n = 5), meningitis TB (n = 2), and skeletal TB (n = 2), and gastrointestinal TB (n = 1). All extraPTB patients had negative chest imaging and no microbiological or radiological evidence of concurrent pulmonary TB.

The primary objective of this study was to analyze and compare differences in COVID-19 manifestations between these two groups.

### 2.2. Inclusion Criteria

Patients who met the following criteria were included in this study:

✓ Confirmed PTB or extraPTB diagnosis within one month prior to *SARS-CoV-2* infection, verified through solid or liquid cultures or GeneXpert test at the TB ambulatory service in Timișoara [19].
✓ *SARS-CoV-2* infection confirmed by RT-PCR from nasopharyngeal swabs at an accredited laboratory [20]. Moderate or severe *SARS-CoV-2* infection at hospital admission. A moderate case was defined by clinical signs of lower respiratory disease and SpO_2_ ≥ 94% on room air, while severe cases presented SpO_2_ < 94%, a respiratory rate > 30 breaths/min, or lung infiltrates > 50% [20].
✓ Normal renal function (diuresis, eGFR, creatinine, and urea within normal limits) [21].
✓ History of BCG vaccination: This criterion was applied to minimize immunological variability and ensure consistency across the cohort, as BCG vaccination may influence host immune responses to MTB and is nearly universally administered in our region [22].

### 2.3. Exclusion Criteria

Patients were excluded if they met any of the following criteria:

× Diagnosed with both pulmonary and extrapulmonary tuberculosis simultaneously.
× Presence of overweight (BMI 25–30 kg/m^2^) or obesity (BMI ≥ 30 kg/m^2^) [23].
× Pre-existing severe or uncontrolled arterial hypertension [24].
× Diagnosed with chronic heart failure, neoplasms, other chronic pulmonary, hepatic, renal, or digestive diseases, HIV infection, or primary or secondary immunodeficiency [25,26,27]. Uncontrolled T2DM was defined as presenting a HbA1c ≥ 8.5% in titration in the last 3 months, the presence of frequent glycemic decompensations (hyperosmolar state, ketoacidosis), or advanced diabetes-related complications (nephropathy, neuropathy, retinopathy) in accordance with ADA recommendations for poor glycemic control [28]. Patients with uncontrolled COPD, defined according to GOLD 2024 criteria (frequent exacerbations, GOLD stage III–IV, or need for long-term oxygen therapy), were excluded due to the potential confounding effect on inflammatory markers and respiratory outcomes [29]. Exclusion criteria were applied to minimize confounding effects on inflammatory markers and COVID-19 severity, ensuring a clearer analysis of clinical differences between PTB and extraPTB patients.

### 2.4. Ethical Considerations

This study was approved by the Ethics Council for Scientific Research at the Victor Babeș University of Medicine and Pharmacy Timișoara, in accordance with the Helsinki Declaration (04/19 January 2021). Written informed consent was obtained from all participants before data collection.

### 2.5. Data Collection

Medical records were reviewed to extract anamnestic, clinical, laboratory, and imaging data.

Medical history included the following:

- BCG vaccination status.
- Comorbidities. Subjects with controlled T2DM or COPD were included, as these are frequent comorbidities in TB and COVID-19, and their presence allowed for evaluation of potential associations with clinical outcomes.
- Smoking status (never smoked/smoker).
- History of previous TB treatment and cure status.

Clinical parameters recorded:

- Body Mass Index, calculated as weight (kg)/height^2^ (m^2^) [30].
- SpO_2_ (%) at *SARS-CoV-2* diagnosis and lowest recorded value during hospitalization [31].
- Peripheral systolic and diastolic blood pressure (SBP, DBP, mmHg) at diagnosis.
- COVID-19 symptoms, including fever, cough, dyspnea, fatigue, abdominal pain, chest pain, myalgia, vomiting, diarrhea, headache, and anosmia/ageusia. Symptoms were classified as severe if associated with tachypnea (respiratory rate ≥ 30 breaths/min) [32].

Laboratory and Imaging Evaluations [33,34,35,36,37,38,39]

- C-reactive protein (CRP, mg/L).
- Procalcitonin (PCT, ng/mL).
- Aspartate aminotransferase (AST, U/L) and alanine aminotransferase (ALT, U/L).
- Lactate dehydrogenase (LDH, U/L).
- Interleukin-6 (IL-6, pg/mL).
- D-dimer levels (mg/L).
- Neutrophil, lymphocyte, and platelet counts (/µL).
- Neutrophil-to-lymphocyte ratio (NLR), platelet-to-lymphocyte ratio (PLR), and systemic immuno-inflammatory index (SII) [33,34,35].
- CT scan interpretation: An experienced radiologist (≥10 years of experience) analyzed chest CT scans at the time of *SARS-CoV-2* infection confirmation. Lung involvement was assessed using a semi-quantitative CT severity score (0–25 points), based on the extent of ground-glass opacifications, consolidations, crazy paving patterns, and other abnormalities [36,37,38,39].

Outcome Measures

- Hospitalization duration (days).
- Symptom severity.
- Resolution (PCR-negative or discharge) or mortality

### 2.6. Statistical Analysis

Data were collected using Microsoft Excel and analyzed with DATAtab: Online Statistics Calculator (DATAtab e.U., Graz, Austria). Normality was assessed with the Shapiro–Wilk test. A significance threshold of *p* = 0.05 was set, and, due to the small study groups, interpretation was based on the Exact *p* value. For group comparisons, Mann–Whitney tests were used for non-normally distributed variables, while *t*-tests were applied for normally distributed variables. Spearman correlation analysis was performed to examine relationships between parameters. AUC-ROC analysis assessed the ability of clinical and laboratory parameters to distinguish between PTB and extraPTB in COVID-19 patients. Logistic regression models were used to identify independent predictors of disease severity and outcomes. Fisher’s exact test evaluated associations between categorical variables. Multicollinearity was checked using Variance Inflation Factors (VIFs), with VIF < 5 considered acceptable. When VIF ≥ 5, a one-way analysis of covariance (ANCOVA) and LASSO regression were applied to mitigate multicollinearity, allowing for the selection of the most relevant predictors of hospitalization duration. LASSO regression penalized less significant variables by reducing their coefficients to zero. A Random Forest classification analysis identified key predictors of fatality. Due to class imbalance, the model was trained with balanced class weights to account for the rarity of fatal cases. While it demonstrated high accuracy in identifying recovered patients, it misclassified one fatal case, highlighting the difficulty of predicting rare events.

### 2.7. Study Limitations

- The retrospective, cross-sectional design limited causal inference.
- The small sample size may impact the statistical power of subgroup analyses.
- While our strict exclusion criteria reduced potential confounders affecting inflammatory markers and outcomes, they also limited the generalizability of our findings. As such, the results are most applicable to a relatively homogeneous patient population with fewer comorbidities. Future studies with broader inclusion criteria are needed to assess external validity.
- We did not evaluate the duration between the first positive and first negative *SARS-CoV-2* RT-PCR due to incomplete data availability. Several patients were discharged or transferred before documentation of swab negativation, which limited consistency in this variable.

## 3. Results

This study included 55 patients with TB–*SARS-CoV-2* co-infection, divided in two study groups according to the nature of TB infection: 32 patients with PTB and 23 patients with extraPTB.

Male patients showed a predominance, with 65% of the cases in the PTB group and 56.5% of cases in the extraPTB group. Regarding age, a higher proportion of young individuals (65% under 40 years old) was noted in the extraPTB group, while 93.8% were over 40 years old in the PTB group. Thus, the mean age was significantly higher in the PTB group (62.8 years, SD = 12.820 versus the extraPTB group (40.1 years, SD = 18.6), *p* < 0.001 (*t*-test). See Table 1.

The comparison revealed significant differences between the two groups in most of the analyzed parameters.

In comparison to the extraPTB-*SARS-CoV-2* group, the PTB group revealed a significantly lower SpO_2_ both at diagnosis and with regard to the lowest value registered, a significantly higher chest CT involvement median score than the extraPTB group (revealing a clear higher pulmonary involvement) and significantly higher SBP and DBP median values. Although both groups had median BMI values within the normal range, the PTB-*SARS-CoV-2* group showed significantly lower BMI values.

With regard to inflammation markers, the data show a higher CRP in the PTB group and a higher IL-6 in the extraPTB group. Procalcitonin did not show significant differences between groups, *p* = 0.09 (Fisher’s Exact test). While both groups exhibited lymphopenia, the extraPTB group displayed significantly lower values. In addition, the PTB group also presented neutropenia. While NLR and PLR were significantly higher in the extraPTB group, SII was significantly higher in the PTB group. No differences were detected between transaminase, LDH, and d-dimer levels. See Table 2.

No significant differences were detected between the two groups with regard to sex, smoking habits, comorbidities such as COPD and T2DM, the severity of symptoms, and outcome (Table 3).

A Random Forest classification analysis was conducted to identify key predictors of symptom severity (mild or severe) among the two groups (Table 4). The model achieved perfect classification, correctly identifying all mild and severe cases, suggesting strong predictive capability (Table 5). The feature importance analysis revealed that oxygenation status was the strongest determinant of symptom severity. The lowest SpO_2_ (importance = 0.19) was the most influential predictor, followed by SpO_2_ at diagnosis (importance = 0.15), highlighting the critical role of hypoxemia in determining symptom severity. Additionally, age (importance = 0.08) and CT involvement score (importance = 0.07) were strong contributors, reinforcing that older patients with greater lung involvement were more likely to experience severe symptoms. Lymphocyte count (importance = 0.06) also emerged as an important factor, potentially reflecting the immune response’s role in disease severity.

The Spearman correlation analysis identified several significant associations among TB type, inflammatory markers, oxygenation parameters, and clinical outcomes. TB type was negatively correlated with mortality (ρ = −0.28, *p* = 0.04), indicating that patients with extraPTB had a lower risk of death compared to those with PTB. Additionally, TB type showed a strong negative correlation with prior TB episodes (ρ = −0.90, *p* < 0.001), suggesting that PTB cases were more frequently associated with tuberculosis reactivation. ExtraPTB patients had significantly longer hospital stays compared to extraPTB patients (ρ = 0.73, *p* < 0.001). PTB patients exhibited lower oxygen saturation at diagnosis (ρ = 0.53, *p* < 0.001) as well as worse oxygenation throughout their illness (ρ = 0.48, *p* < 0.001), suggesting more severe respiratory impairment in PTB cases. CT involvement scores were also significantly higher in PTB patients (ρ = −0.77, *p* < 0.001), reflecting greater pulmonary damage. Additionally, inflammatory markers such as NLR (ρ = 0.66, *p* < 0.001) and SII (ρ= 0.66, *p* < 0.001) were strongly associated with PTB, suggesting a heightened inflammatory response. PCT positivity was correlated with increased mortality risk (ρ = 0.31, *p* = 0.02), while D-dimer levels showed a significant association with prolonged hospitalization (ρ = 0.28, *p* = 0.04). These findings underscore the distinct clinical trajectories of PTB and extraPTB in *SARS-CoV-2* co-infection, with PTB patients exhibiting more severe disease manifestations, greater inflammation, and poorer respiratory function. See Table A1 in Appendix A.

**Figure 1:**
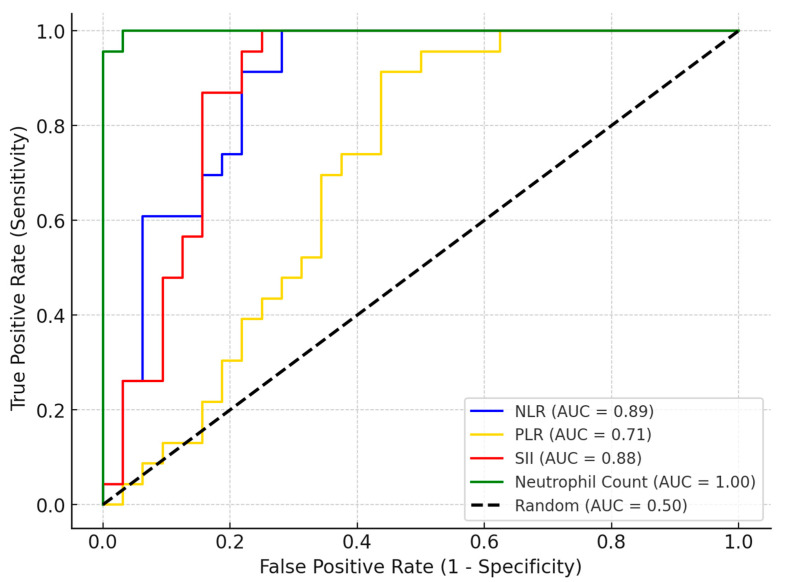
AUC-ROC curves illustrating the ability of inflammatory and hematologic markers (neutrophil count, NLR, PLR, SII) to discriminate between PTB and extraPTB in *SARS-CoV-2* co-infection.

**Figure 2:**
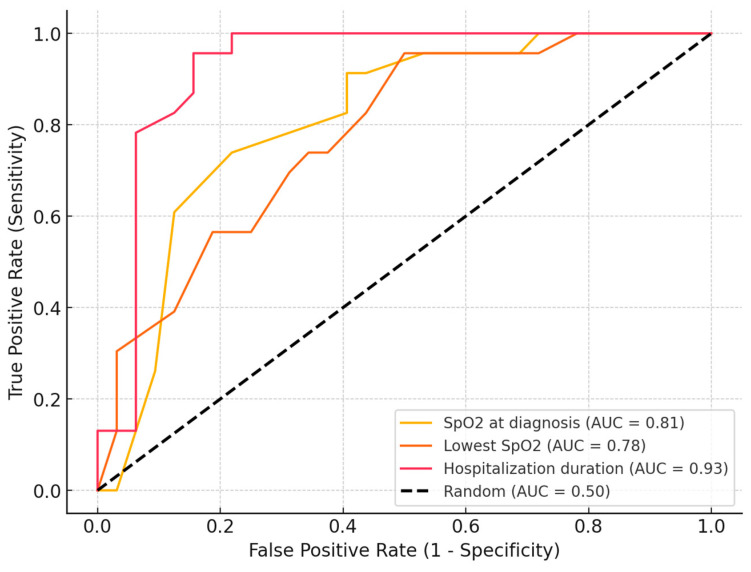
AUC-ROC curves showing the ability of hospitalization duration and oxygenation parameters (SpO_2_ at diagnosis and lowest recorded SpO_2_) to distinguish between TB localization groups.

The AUC-ROC analysis evaluated the ability of multiple clinical and laboratory parameters to distinguish extraPTB from PTB cases (Table 6). Among the evaluated markers, neutrophil count emerged as the most effective discriminator, with an optimal cutoff of 3300 cells/µL, yielding 100% sensitivity and 96.88% specificity. Additionally, inflammatory markers such as NLR (AUC = 0.89), SII (AUC = 0.88), and PLR (AUC = 0.71) showed strong predictive value, achieving high sensitivity while maintaining moderate specificity (Figure 1). SpO_2_ at diagnosis also demonstrated a high discriminatory power (AUC = 0.81), while the lowest SpO_2_ registered showed strong predictive ability (AUC = 0.78), achieving a high sensitivity but a lower specificity. Hospitalization duration also demonstrated strong discriminatory ability (AUC = 0.93), with an optimal cutoff of 20 days, yielding 95.65% sensitivity and 84.38% specificity (Figure 2). This suggests that longer hospital stays are strongly associated with extraPTB cases, distinguishing them from PTB cases with high accuracy. Several markers, including the status of associating COPD and type 2 DM, prior TB history, the presence of severe symptoms, CRP, procalcitonin, CT involvement score, and TB outcome, exhibited 0% sensitivity, meaning that no extraPTB cases were correctly identified based on the chosen cutoff. This suggests that either these markers lack discriminatory power for distinguishing extraPTB or that the selected cutoffs (determined by Youden’s index) are too restrictive. In the case of CT involvement scores, PTB cases consistently showed higher values, which may have biased the optimal threshold toward excluding extraPTB cases. Similarly, markers such as COPD and PCT levels may not have sufficient variation between the two groups to serve as reliable classifiers.

To minimize the effect of covariates and further explore the effect of TB type (PTB vs. extraPTB) on hospitalization duration, an ANCOVA was conducted while adjusting for multiple clinical and laboratory parameters. The overall model was statistically significant (F = 12.71, *p* < 0.001), explaining approximately 85.4% of the variance in hospitalization duration (adjusted R^2^ = 0.787). Interestingly, although previous results have shown that extraPTB subjects presented a significantly longer hospitalization, the ANCOVA showed that TB type did not significantly influence hospitalization duration (F = 1.17, *p* = 0.287) when controlling for other factors. Among the covariates, a higher BMI was associated with shorter hospitalization (F = 9.1, *p* = 0.005), whereas inflammatory and coagulation markers were significant predictors of prolonged hospitalization. Increased levels of IL-6 (F = 5.21, *p* = 0.02), D-dimer (F = 8.02, *p* = 0.007), neutrophil count (F = 15.22, *p* < 0.001), and SII (F = 4.83, *p* = 0.034) were all strongly associated with extended hospital stays. These results suggest that inflammatory markers as covariates were more powerful that TB type in determining longer hospital stay. Additionally, CT involvement score and CRP levels approached statistical significance, suggesting a potential role in hospitalization duration (Table 7).

After performing the ANCOVA, a LASSO regression analysis was performed to identify the most influential predictors of hospitalization duration while addressing multicollinearity. The final model retained five key predictors, while other variables were eliminated due to minimal contribution. The most influential predictor was the neutrophil count (β = 6.53), indicating that higher neutrophil levels were strongly associated with prolonged hospitalization. TB type remained in the model (β = 4.71), suggesting a potential, though moderate, association with hospitalization length. IL-6 levels (β = 4.04) were also retained, reinforcing the role of systemic inflammation in prolonged hospital stays. In contrast, platelet count (β = −3.69) and BMI (β = −2.60) were negatively associated with hospitalization duration, suggesting that higher platelet levels and increased BMI were linked to shorter hospital stays. All other variables, including SpO_2_ levels, CRP, D-dimer, and CT involvement score, were removed from the model, indicating that they had little independent predictive value after adjusting for stronger correlates (Table 8).

Out of the 55 subjects, a number of 7 in the PTB group (all men) and 1 (a woman) in the extraPTB group had fatal outcomes (14.5% from all subjects). Five of these subjects were smokers, six also had T2DM, and 3, COPD. All subjects presented severe symptoms at presentation and positive procalcitonin. Table 9 shows the median values of the paraclinical parameters in these subjects.

Regarding the predictors of fatality, a penalized logistic regression was performed as an approximation of Firth’s logistic regression to identify predictors of fatal outcomes among the two groups. Given the small number of fatal cases (8 cases), a regularized approach was applied to stabilize coefficient estimates. The results indicate that a younger age (OR = 0.58, 95% CI: 0.44–0.75) and a higher value of the lowest SpO_2_ registered (OR = 0.57, 95% CI: 0.44–0.74) are the strongest predictors of lower fatality risk. However, initial SpO_2_ at diagnosis (OR = 1.23, 95% CI: 0.95–1.60) showed a positive association with fatality, meaning that patients with relatively higher initial oxygen levels may have still experienced worsening conditions leading to mortality. This could indicate delayed deterioration in severe TB-COVID cases. TB type was not a significant predictor of fatality (OR = 0.99, 95% CI: 0.76–1.28). BMI (OR = 0.94, 95% CI: 0.73–1.23) also did not show a significant effect. See Table A2, in the Appendix A.

In order to further explore predictors of fatal outcome, a Random Forest classification analysis was performed (Table 10). The classification model achieved high accuracy in identifying healed cases but misclassified one fatal case, reflecting the challenge of predicting rare events (Table 11). The feature importance analysis showed that CT involvement score was the most influential predictor of mortality (importance = 0.22), suggesting that greater lung involvement significantly increases the risk of death. Oxygenation status was also a major determinant of fatal outcomes, with the lowest SpO_2_ (importance = 0.13) ranking as the second most important predictor, followed by SpO_2_ at diagnosis (importance = 0.09). This highlights severe hypoxemia and respiratory failure as key contributors to mortality. In addition, systemic inflammation and tissue damage were strong predictors of fatality, as indicated by CRP (importance = 0.08) and LDH (importance = 0.06). Notably, TB type was not among the top-ranked predictors of fatality, further supporting the finding that mortality risk was driven more by disease severity and systemic inflammation rather than TB localization.

## 4. Discussion

*SARS-CoV-2* and tuberculosis co-infection presents intricate clinical challenges, particularly when differentiating between pulmonary TB and extrapulmonary TB. Our study, involving 55 subjects, unveiled significant distinctions between these two groups, focusing on the differences in clinical presentation, biomarkers, imagistic findings, and disease outcomes. Despite the small samples, this analysis is pertinent in expanding knowledge on the subject. This study is built on our previous work [17,18] regarding the analysis of the manifestations of *SARS-CoV-2* infection alone versus *SARS-CoV-2*–PTB and extraPTB co-infection, respectively. To our knowledge, there are no other publications comparing the two categories of co-infection, and, especially with regard to extraPTB and COVID-19 co-infection, there is a significant research gap.

### 4.1. Demographic Characteristics

In our cohort, males predominated in both PTB (65%) and extraPTB (56.5%) groups. Globally, tuberculosis affects significantly more men than women. A meta-analysis of 29 surveys across 14 countries confirmed a consistent male bias in both notification and prevalence rates [40]. Higher TB rates and more severe disease in men result from a combination of healthcare access, biological, and behavioral factors. Some studies suggest gender-based disparities in healthcare access [41], but research from Mexico and South India indicates that women seek medical care more frequently than men [42,43]. Men often delay seeking care, as symptoms like coughing may be attributed to tobacco use, leading to longer symptomatic periods before diagnosis and more severe disease at presentation. Biologically, TB progression rates vary by sex and age, with women of reproductive age at higher risk of progression, while men experience increased susceptibility as they age [44,45]. Additionally, co-morbidities such as HIV, diabetes, and cirrhosis—more prevalent in men—can accelerate TB progression [46,47,48]. Men are also more exposed to high-risk environments, including prisons, shelters, and poorly ventilated workplaces [49,50,51], and outbreaks have been linked to clandestine bars with predominantly male customers [52]. Furthermore, alcohol and tobacco use, more common among men, contribute to faster TB progression and worse treatment outcomes, even after adjusting for alcohol consumption [53,54]. These factors highlight the need for targeted public health interventions focused on early detection and prevention strategies for high-risk male populations.

Epidemiological data on sex differences in extraPTB remain inconclusive. A study in Mali analyzing 1012 confirmed cases reported a male-to-female ratio of 1.59:1, with men more likely to develop pleural TB and women having higher odds of lymph node and abdominal TB [55]. In contrast, studies from Korea and Madrid found a greater prevalence of extraPTB among women, particularly those aged 40–60 [3,56].

Age is another critical risk factor for TB [57] and severe COVID-19 [58]. In our study, differences were significant between the two groups, with PTB subjects presenting a higher mean age (62.8 years) compared to the extraPTB subjects (40.1 years). The Random Forest classification identified older age as an important predictor of severe symptoms. Moreover, Firth’s logistic regression showed that younger age is a predictor of lower fatality risk (OR = 0.58). Several studies have highlighted that older individuals are at a significantly higher risk of severe disease and mortality. A study involving 89 co-infected adults found a mean age of 45.14 years, with 75.3% being male [59], while a Ugandan study described 11 cases with a mean age of 46.9 years [60]. However, increasing age has been consistently associated with worsening outcomes. In a U.S. cohort of 333 patients with COVID-19 and TB co-infection, mortality risk increased significantly with age; individuals aged 45–64 had an adjusted prevalence ratio (aPR) of 5.6, those aged 65–74 had an aPR of 8.6, while those over 85 exhibited a strikingly high aPR of 25 [61]. Another study of 153 hospitalized co-infected patients reported that individuals aged 65 and above had a significantly higher likelihood of severe disease, with an OR of 9.55 [62]. These findings suggest that older individuals not only experience more severe disease progression but also have increased mortality risks, emphasizing the need for enhanced monitoring, early intervention, and targeted therapeutic strategies in this demographic.

Younger individuals are more predisposed to developing extraTB due to their relatively stronger immune responses, whereas older adults are more susceptible to PTB [63]. Young adults with robust cell-mediated immunity are more likely to confine the infection to specific extrapulmonary sites, resulting in localized manifestations such as lymph node, pleural, or bone TB. In contrast, although elderly individuals may experience more disseminated forms of TB, their overall likelihood of developing extraPTB is lower compared to younger populations [64]. As a result, pulmonary TB is more frequently observed in older individuals, often due to the reactivation of latent infections, whereas extraPTB is more common among younger adults, particularly in regions with a high TB burden [11]. This pattern could also explain the observed age disparity between groups in our study.

### 4.2. Key Findings Regarding Clinical and Inflammatory Parameters

In our analysis, although both groups had median BMI values within the normal range, the PTB-*SARS-CoV-2* group showed significantly lower BMI values, which may suggest relative undernutrition. A higher BMI was linked to shorter hospital stays. A low BMI is a well-established risk factor for adverse TB outcomes, affecting both PTB and extraPTB subjects. Malnutrition and low BMI have been linked to impaired immune function, increasing susceptibility to MTB infection and disease progression in both forms of TB [65,66,67,68]. Studies have shown that underweight individuals with TB, regardless of disease localization, have higher bacterial loads, delayed sputum culture conversion (in PTB), and poorer treatment outcomes, including increased mortality rates [69]. The immunosuppressive effects of malnutrition may exacerbate systemic inflammation and hinder granuloma formation, weakening the body’s ability to contain both pulmonary and extrapulmonary TB infections. Additionally, patients with low BMI frequently experience prolonged hospitalization and higher relapse rates after treatment completion [70]. In extraPTB, malnutrition can contribute to more extensive disseminated disease, lymphatic involvement, and increased risk of central nervous system TB, including tuberculous meningitis, which carries a high fatality rate [69,70]. Given these findings, nutritional interventions should be considered a critical component of TB management, particularly in underweight patients with PTB or extraPTB, to improve treatment efficacy and overall prognosis [69,70]. In contrast, COVID-19 severity is more frequently observed in individuals with overweight or obesity [71,72]. However, frailty, a condition commonly seen in TB patients, is also a significant risk factor for increased mortality and prolonged hospitalization among those with COVID-19 [73]. Frailty is linked to both ends of the BMI spectrum, affecting both underweight individuals [74] and those with severe obesity [75]. Maintaining a healthy BMI is linked to improved outcomes in both acute and chronic diseases [74]. Given the role of underweight status in tuberculosis progression, BMI may still carry prognostic implications; however, it should not be interpreted as inherently worse than higher BMI values known to promote inflammation in COVID-19.

PTB-COVID-19 co-infection manifested in reduced oxygen saturation and higher chest CT involvement scores, indicating greater pulmonary compromise in comparison to extraPTB-COVID-19. The differences in pulmonary manifestations between the two groups were so significant that even the AUC-ROC analysis revealed that a high SpO_2_ levels at diagnosis and a higher value of the lowest value registered represent the strongest predictors of extraPTB, while traditional inflammatory and radiological markers showed limited ability to differentiate between TB subtypes in the context of *SARS-CoV-2* co-infection. PTB and COVID-19 co-infection presents a significant challenge due to overlapping inflammatory pathways. PTB primarily relies on a Th1-mediated immune response, with IFN-γ and TNF-α playing essential roles in bacterial containment [76]. However, *SARS-CoV-2* infection disrupts immune homeostasis, leading to lymphopenia and reduced CD4+ T-cell responses, which can impair MTB control and increase the risk of reactivation [77]. In severe COVID-19 cases, the cytokine storm, marked by increased IL-6, TNF-α, and IFN-γ expression, exacerbates lung inflammation and worsens TB disease, heightening the risk of acute respiratory distress syndrome and respiratory failure, often necessitating intensive care and mechanical ventilation [78]. In contrast, extraPTB is characterized by a stronger regulatory T-cell response, which may help limit pulmonary damage but allows persistent MTB infection. Unlike PTB, extraPTB-COVID-19 is less likely to cause severe pulmonary complications, though it may lead to increased systemic inflammation due to IL-10 and IL-4 upregulation [79,80]. While patients with extraPTB generally have preserved lung function, those with TB meningitis or disseminated TB face significantly higher mortality rates [70].

A Random Forest classification analysis was conducted to identify key predictors of symptom severity (moderate or severe) among the two groups. The feature importance analysis revealed that oxygenation status was the strongest determinant of symptom severity: the lowest SpO_2_ registered was the most influential predictor, followed by SpO_2_ at diagnosis, highlighting the critical role of hypoxemia in determining symptom severity. Additionally, older age, a higher CT involvement score, and a low lymphocyte count were also strong contributors. These results reinforce the knowledge that older patients with greater lung involvement were more likely to experience severe symptoms.

Inflammatory markers differed between groups: CRP levels were higher in PTB-*SARS-CoV-2* patients, while IL-6 levels were elevated in extraPTB patients. Both groups experienced lymphopenia, but it was more pronounced in the extraPTB group. These findings align with existing research highlighting the role of inflammatory markers in assessing TB severity and monitoring treatment response [62].

Our previous study [18] demonstrated that, while individuals with extraPTB-*SARS-CoV-2* co-infection may exhibit milder respiratory symptoms, they remain at risk for severe systemic inflammation, necessitating close monitoring. Notably, elevated levels of PCT and IL-6 have been recognized as key indicators of severe COVID-19 outcomes, highlighting the need for further investigation into their significance in co-infected patients. Several biomarkers associated with COVID-19 progression and the onset of acute respiratory distress syndrome include elevated LDH, CR, IL-6, D-dimer levels, lymphocyte and platelet counts, renal function markers, and high-sensitivity troponin. In addition, critically ill patients frequently exhibit marked lymphopenia and neutrophilia, contributing to an increased neutrophil-to-lymphocyte ratio [81].

These findings confirm that worsening oxygenation, greater lung involvement, and systemic immune response are the primary drivers of severe symptoms in TB-COVID-19 patients. These insights may aid in early risk stratification and targeted interventions for high-risk individuals.

### 4.3. Hospitalization and Mortality

Hospitalization duration in TB-COVID-19 co-infected patients was primarily influenced by inflammatory markers rather than the type of TB (PTB vs. extraPTB), which did not show a significant effect on hospital stay length. Higher levels of IL-6, D-dimer, neutrophil count, and systemic inflammatory index were significantly associated with prolonged hospitalization, while a higher BMI was linked to shorter hospital stays. These findings are in line with other research regarding disease severity and mortality risk [82]. The LASSO regression analysis further confirmed that neutrophil-driven inflammation and systemic IL-6 activation were dominant predictors of hospitalization outcomes, while the potential protective role of higher BMI warrants further investigation, possibly due to differences in metabolic reserves or immune responses.

Mortality risk in TB-COVID-19 patients was most strongly associated with lung involvement, oxygenation status, and systemic inflammation, rather than TB localization. A proportion of 14.5% of subjects had a fatal outcome (12.7% with PTB and 1.8% with extraPTB). CT involvement score emerged as the most critical predictor of fatality, highlighting the significant impact of lung damage on patient outcomes. Oxygenation parameters, including SpO_2_ at diagnosis and the lowest SpO_2_, were the next most influential factors, reinforcing the role of hypoxemia and respiratory failure in driving mortality risk. Additionally, CRP and LDH were key markers of disease severity, emphasizing the role of inflammatory and tissue damage pathways in TB-COVID-19 co-infection.

A Random Forest classification analysis suggested that PTB cases were more likely to be fatal compared to extraPTB, potentially due to higher pulmonary involvement. However, Firth’s logistic regression found no statistically significant association between TB type and mortality risk, indicating that the increased fatality in PTB cases may reflect disease severity rather than TB localization alone. Despite methodological differences between these models, both consistently identified oxygenation impairment (lowest SpO_2_), inflammation (CRP, LDH), and lung involvement (CT score) as the primary predictors of mortality. These findings underscore the need for early identification of high-risk patients based on respiratory parameters and systemic inflammation, rather than TB localization, to optimize treatment strategies and improve survival outcomes.

While the Random Forest models demonstrated perfect classification in predicting symptom severity and high accuracy in predicting fatality, these results should be interpreted cautiously due to the small sample size. In the case of fatality prediction, one fatal case was misclassified, reflecting the difficulty of rare event modeling. The F1-score, precision, and recall were optimal for severity classification but less reliable for mortality outcomes, highlighting the need for larger, balanced datasets to validate these results. The use of Firth’s logistic regression helped correct for bias in rare event estimation, but the wide confidence intervals reflect limited statistical power.

An important publication by the TB/COVID-19 Global Study Group places mortality of the studied patients with TB and COVID-19 at a prevalence of 11.1%. It is important to mention that the study cites different causes of death: 49.4% died from severe COVID-19, 36.5% from COVID-19 and TB co-infection, and 1.2% died from TB only. Among the patients who died for other reasons, 5.9% died with COVID-19 (multiple comorbidities, cancer, sarcoidosis, and HIV), and the remaining 7% died after the resolution of COVID-19 (sepsis, multiple comorbidities, pneumonia, and pulmonary thromboembolism) [83]. A meta-analysis places mortality rates by PTB/COVID-19 co-infection between 7.6% and 23.6%, depending on severity [62]. In extraPTB-COVID-19, prognosis depends on organ involvement, with TB meningitis carrying the highest fatality risk [84].

### 4.4. Implications for Clinical Practice

The distinct clinical trajectories observed between PTB and extraPTB patients co-infected with *SARS-CoV-2* underscore the need for tailored clinical management strategies [85]. Enhanced monitoring and early intervention may be particularly crucial for PTB patients, given their higher risk of severe disease and mortality. The pronounced lymphopenia observed in extraPTB patients suggests a need for the vigilant monitoring of immune status and potential adjustments in immunomodulatory therapies. Patients in need of long-term rehabilitation after disease resolution could benefit from pulmonary rehabilitation alone and in combination with progressive muscular relaxation for improving physical function, mental health, and sleep quality [86].

### 4.5. Study Limitations and Future Directions

While our study provides valuable insights, it is limited by its small sample size and single-center design, which may affect the generalizability of the findings. Future multicenter studies with larger cohorts are warranted to validate these observations and further elucidate the complex interplay between PTB, extraPTB, and COVID-19.

## 5. Conclusions

Our study highlights the complex interplay between tuberculosis and COVID-19 co-infection, with distinct differences in clinical presentation, inflammatory responses, and disease outcomes between pulmonary TB and extrapulmonary TB cases. While extraPTB patients exhibited less severe respiratory involvement, they remained at risk for systemic inflammation.

Key predictors of hospitalization and mortality were identified, demonstrating that TB localization did not independently affect hospital stay length or fatality risk. Instead, oxygenation impairment (lowest SpO_2_, SpO_2_ at diagnosis), inflammatory markers (CRP, LDH, IL-6), and lung involvement (CT score) emerged as the dominant determinants of severe outcomes. The analysis consistently pointed to neutrophil-driven inflammation and systemic IL-6 activation as key contributors to hospitalization duration, while higher BMI was associated with shorter hospital stays, potentially due to metabolic reserves or immune response differences.

Mortality risk was most strongly correlated with hypoxemia and extensive pulmonary involvement, rather than TB type. Despite differences between machine learning models, both consistently identified severe lung involvement and inflammatory burden as the primary drivers of fatality.

These findings reinforce the need for early risk stratification based on oxygenation status, inflammatory markers, and CT involvement scores, rather than TB localization alone. Targeted interventions, including nutritional support for underweight patients, the aggressive management of systemic inflammation, and the enhanced monitoring of oxygenation parameters, are critical in improving outcomes for co-infected individuals. Future research should focus on the long-term implications of TB-COVID-19 co-infection, especially in extraPTB cases, where existing data remain scarce.

In conclusion, our study highlights significant differences between PTB and extraPTB patients co-infected with *SARS-CoV-2*. Recognizing these distinctions is vital for optimizing patient management and improving prognostic assessments in this unique patient population.

## Figures and Tables

**Table 1:** The number of subjects by sex and age.

		PTB-*SARS-CoV-2* n = 32	extraPTB-*SARS-CoV-2* n = 23
Sex	F	11 (34%)	10 (43.5%)
	M	21 (65%)	13 (56.5%)
Age	19–40 y	2 (6.2%)	15 (65.2%)
	41–65 y	16 (50%)	6 (26.1%)
	>66 y	14 (43.8%)	2 (8.7%)

**Table 2:** A Mann–Whitney comparison of the analyzed parameters between PTB-*SARS-CoV-2* and extraPTB-*SARS-CoV-2* groups.

Parameter	PTB-*SARS-CoV-2* Median Value	extraPTB-*SARS-CoV-2* Median Value	*p*
BMI (kg/m^2^)	21.88	24.45	**0.01**
SpO_2_ at diagnosis (%)	90	96	**<0.001**
Lowest SpO_2_ (%)	83.5	92	**0.001**
Peripheral SBP at diagnosis (mmHg)	138	129	**0.03**
Peripheral DBP at diagnosis (mmHg)	92	85	**0.04**
CRP (mg/dL)	89.5	66.1	**0.01**
LDH (UI/L)	288	203	0.14
IL-6 (pg/mL)	4.2	8.8	**0.009**
AST (UI/L)	36	38	0.28
ALT (UI/L)	38.5	42	0.74
D-dimer (mg/L)	1.91	1.65	0.55
Neutrophils/uL *	1539.69 (SD = 933.29)	6194.8 (SD = 1837.6)	**<0.001**
Lymphocytes/uL	2210	1460	**0.04**
Thrombocytes/uL	242,500	351,000	**0.02**
NLR	2	3.73	**<0.001**
PLR	128.85	235.54	**0.01**
SII	134,549.68	1,255,888.88	**<0.001**
Chest CT involvement score	16	6	**<0.001**

* The Student’s *t*-test is employed for neutrophil count. The bolded data in this table represent statistically significant results.

**Table 3:** Fisher’s exact comparison of the nominal parameters between PTB-*SARS-CoV-2* and extraPTB-*SARS-CoV-2* groups.

Parameter	*p*
Sex	0.7
Smoking	0.4
Associating COPD	0.86
Associatiang T2MD	0.82
Severity of symptoms	0.7
Outcome	0.54

**Table 4:** Random Forest classification analysis: Predictors of severe symptoms.

Feature	Importance
Lowest SpO_2_	0.20
SpO_2_ at diagnosis	0.15
Age	0.08
CT involvement score	0.07
Lymphocyte count	0.07
LDH	0.06
CRP	0.06
D-dimer	0.05
IL6	0.05
Neutrophil count	0.04
AST	0.03
Thrombocyte count	0.03
ALT	0.03
BMI	0.02
PLR	0.02
SII	0.02
NLR	0.02
Type of TB	0.01

**Table 5:** Random Forest classification report for predictors of severe symptom analysis.

	Precision	Recall	F1-Score	Support
Mild symptoms	1	1	1	38
Severe symptoms	1	1	1	17
Accuracy	1	1	1	1
Macro-average	1	1	1	55
Weighted average	1	1	1	55

**Table 6:** AUC-ROC analysis: The ability of clinical and paraclinical markers to detect extraPTB cases.

Parameter	AUC	Criterion (Cut-Off)	*p*-Value	Sensitivity %	Specificity %	PPV	NPV
BMI	0.7	23.45	0.06	65.22	84.38	0.75	0.77
SpO_2_ at diagnosis	0.81	95	0.01	73.91	78.12	0.71	0.81
Lowest SpO_2_	0.78	84	0.03	95.65	50	0.58	0.94
AST	0.59	74	0.62	34.78	100	1	0.68
ALT	0.53	95	0.38	21.74	100	1	0.64
LDH	0.38	166	0.09	86.96	25	0.45	0.73
IL6	0.71	8.8	0.07	52.17	84.38	0.71	0.71
D-dimer	0.55	4.86	0.12	26.09	100	1	0.65
Neutrophil count	0.95	3300	<0.001	100	96.88	0.96	1
Lymphocyte count	0.34	810	0.23	91.3	15.62	0.44	0.71
Thrombocyte count	0.68	242,000	0.08	91.30	50.00	0.57	0.89
NLR	0.89	1.57	0.02	100	71.88	0.72	1
PLR	0.71	154.95	0.06	91.3	56.25	0.6	0.9
SII	0.88	437,758.9	0.04	100	75	0.74	1
Hospitalization	0.93	20	0.02	95.65	84.38	0.81	0.96

**Table 7:** ANCOVA of the predictors of hospitalization duration.

	Sum of Squares	df	F	*p*
Type of TB	50.62	1	1.53	0.22
Age	105.41	1	3.18	0.08
BMI	305.41	1	9.21	**0.004**
SpO_2_ at diagnosis	2.05	1	0.06	0.81
Lowest SpO_2_	19.89	1	0.60	0.44
CRP	79.45	1	2.39	0.13
AST	9.57	1	0.29	0.59
ALT	1.08	1	0.03	0.86
LDH	4.76	1	0.14	0.71
IL6	151.40	1	4.56	**0.04**
D-dimer	287.69	1	8.67	**0.005**
Neutrophil count	524.87	1	15.82	**0.0003**
Thrombocyte count	24.06	1	0.73	0.4
Lymphocyte count	102.70	1	3.10	0.08
CT_involvement_score	100.32	1	3.02	0.09
NLR	79.10	1	2.38	0.13
PLR	108.50	1	3.27	0.08
SII	181.05	1	5.46	**0.03**
Residual	1194.30	36		

The bolded data in this table represent statistically significant results.

**Table 8:** LASSO regression of the most influential predictors of hospitalization duration while addressing multicollinearity.

Variable	β Coefficient
Neutrophil count	6.526728
Type of TB	4.713506
IL6	4.039135
Thrombocyte count	−3.68729
BMI	−2.59693
CT involvement score	2.152821
D-dimer levels	1.903791
SII	−1.77909
CRP	−1.45796
LDH	1.201368
AGE	−0.74844
AST	0.699141
PLR	−0.14857
SpO_2_ at diagnosis	0
Lowest SpO_2_	0
ALT	0
Lymphocyte count	0
NLR	0

**Table 9:** Median values of paraclinical parameters in the subjects with fatal outcomes.

Age	BMI	SpO_2_ at Diagnosis	Lowest SpO_2_
61	21.22	89	79
CRP	IL6	LDH	D-dimer
112	9.1	388	3.31
Neutrophil count	Lymphocyte count	Thrombocyte count	CT involvement score
2910	1000	380,000	22
NLR	PLR	SII	Hospitalization
2.88	333.33	717,250	18

**Table 10:** Random Forest classification analysis: Predictors of fatal outcome.

Feature	Importance
CT involvement score	0.22
Lowest SpO_2_	0.13
SpO_2_ at diagnosis	0.09
CRP	0.08
LDH	0.06
Lymphocyte count	0.06
IL6	0.06
Age	0.04
BMI	0.04
PLR	0.04
Neutrophil count	0.03
NLR	0.03
D-dimer	0.03
SII	0.03
AST	0.02
Type of TB	0.01
Thrombocyte count	0.01
ALT	0.01

**Table 11:** Random Forest classification report for predictors of fatal outcome analysis.

	Precision	Recall	F1-Score	Support
Resolution	0.9	1	0.94	9
Fatality	1	0.5	0.66	2
Accuracy	0.9	0.9	0.9	0.9
Macro-Average	0.95	0.75	0.8	11
Weighted Average	0.91	0.9	0.89	11

## Data Availability

The findings of this study are based on data that can be made available upon request from the corresponding author, though ethical restrictions prevent public disclosure.

## Abbreviations

ADA	American Diabetes Association
ALT	Alanine aminotransferase
ANCOVA	One-way analysis of covariance
Apr	Adjusted prevalence ratio
AST	Aspartate aminotransferase
AUC	Area under the receiver operating characteristic curve
BMI	BMI, Body Mass Index
COPD	Chronic Obstructive Pulmonary Disease
COVID-19	Coronavirus Disease 2019
CRP	C-reactive protein
CT	Computer tomography
DM	Diabetes Mellitus
extraPTB	Extrapulmonary tuberculosis
GOLD	Global Initiative for Chronic Obstructive Lung Disease
HIV	Human Deficiency Virus
IL-6	Interleukin 6
LASSO regression	Least Absolute Shrinkage and Selection Operator regression
LDH	Lactate dehydrogenase
*n*	Number of subjects
MTB	Mycobacterium tuberculosis
OR	Odds ratio
*p*	*p*-value
PCT	Procalcitonin
PTB	pulmonary tuberculosis
ROC	Receiver operating characteristic
RT-PCR	Reverse Transcription Polymerase Chain Reaction
SpO_2_	Saturation of peripheral oxygen
*SARS-CoV-2*	Severe Acute Respiratory Syndrome Coronavirus 2
TB	Tuberculosis
WHO	World Health Organization

## Appendix A

**Table A1:** Spearman correlations between parameters.

	Type of TB	Outcome	Smoking	COPD	T2DM	Prior TB	PCT	NLR	PLR	SII	D -Dimer	Hospitalization	SpO_2_ at dg	Lowest SpO_2_	CT Score
Type of TB	1	−0.28	0.1	−0.02	−0.27	−0.90	−0.07	0.66	0.35	0.66	0.08	0.73	0.53	0.48	−0.77
*p*	0	0.04	0.45	0.9	0.05	<0.001	0.6	<0.001	0.008	<0.001	0.56	<0.001	<0.001	0.0002	<0.001
Outcome	−0.28	1	0.12	0.17	0.39	0.32	0.31	0.17	0.34	0.13	0.28	0.001	−0.45	−0.50	0.61
*p*	0.04	0	0.37	0.2	0.003	0.02	0.02	0.22	0.01	0.34	0.04	0.99	<0.001	<0.001	<0.001
Smoking	0.10	0.12	1	0.36	−0.11	−0.16	−0.07	0.13	0.08	0.08	0.14	0.21	−0.12	−0.03	−0.04
*p*	0.45	0.37	0	0.006	0.44	0.25	0.6	0.33	0.57	0.57	0.32	0.12	0.39	0.8	0.79
COPD	−0.02	0.17	0.36	1	0.13	0.07	−0.06	0.10	0.02	0.04	0.22	0.14	−0.17	−0.22	0.04
*p*	0.9	0.2	0.006	0	0.33	0.62	0.67	0.48	0.88	0.76	0.11	0.31	0.21	0.1	0.78
T2DM	−0.27	0.39	−0.11	0.13	1	0.33	0.39	−0.01	0.11	−0.04	0.23	−0.06	−0.47	−0.46	0.36
*p*	0.05	0.003	0.44	0.33	0	0.01	0.003	0.94	0.42	0.79	0.09	0.65	<0.001	<0.001	0.007
Prior TB	−0.90	0.32	−0.16	0.07	0.33	1	0.14	−0.52	−0.22	−0.53	−0.006	−0.66	−0.54	−0.51	0.75
*p*	<0.001	0.02	0.25	0.62	0.01	0	0.32	<0.001	0.11	<0.001	0.96	<0.001	<0.001	<0.001	<0.001
PCT	−0.07	0.31	−0.07	−0.06	0.39	0.14	1	0.33	0.48	0.37	0.44	0.04	−0.47	−0.53	0.19
*p*	0.60	0.02	0.60	0.67	0.003	0.32	0	0.01	<0.001	0.005	<0.001	0.79	<0.001	<0.001	0.16
NLR	0.66	0.17	0.13	0.10	−0.01	−0.52	0.33	1	0.77	0.95	0.45	0.62	−0.0016	−0.05	−0.29
*p*	<0.001	0.22	0.33	0.48	0.94	<0.001	0.01	0	<0.001	<0.001	<0.001	<0.001	0.99	0.72	0.03
PLR	0.35	0.34	0.08	0.02	0.11	−0.22	0.48	0.77	1	0.86	0.66	0.26	−0.38	−0.42	−0.05
*p*	0.008	0.01	0.57	0.88	0.42	0.11	<0.001	<0.001	0	<0.001	<0.001	0.06	0.004	0.001	0.73
SII	0.66	0.13	0.08	0.04	−0.04	−0.53	0.37	0.95	0.86	1	0.54	0.54	−0.05	−0.11	−0.32
*p*	<0.001	0.34	0.57	0.76	0.79	<0.001	0.005	<0.001	<0.001	0	<0.001	<0.001	0.70	0.41	0.02
D-dimer	0.08	0.28	0.14	0.22	0.23	−0.006	0.44	0.45	0.66	0.54	1	0.14	−0.42	−0.48	0.07
*p*	0.56	0.04	0.32	0.11	0.09	0.96	<0.001	<0.001	<0.001	<0.001	0	0.31	0.001	<0.001	0.62
Hospitalization	0.73	0.001	0.21	0.14	−0.06	−0.66	0.04	0.62	0.26	0.54	0.14	1	0.35	0.29	−0.36
*p*	<0.001	0.99	0.12	0.31	0.65	<0.001	0.79	<0.001	0.06	<0.001	0.31	0	0.009	0.03	0.007
SpO_2_ at dg	0.53	−0.45	−0.12	−0.17	−0.47	−0.54	−0.47	−0.0016	−0.38	−0.05	−0.42	0.35	1	0.95	−0.57
*p*	<0.001	<0.001	0.39	0.21	<0.001	<0.001	<0.001	0.99	0.004	0.70	0.001	0.009	0	<0.001	<0.001
Lowest SpO_2_	0.48	−0.50	−0.03	−0.22	−0.46	−0.51	−0.53	−0.05	−0.42	−0.11	−0.48	0.29	0.95	1	−0.52
*p*	<0.001	<0.001	0.8	0.1	<0.001	<0.001	<0.001	0.72	0.001	0.41	<0.001	0.03	<0.001	0	<0.001
CT score	−0.77	0.61	−0.04	0.04	0.36	0.75	0.19	−0.29	−0.05	−0.32	0.07	−0.36	−0.57	−0.52	1
*p*	<0.001	<0.001	0.79	0.78	0.007	<0.001	0.16	0.03	0.73	0.02	0.62	0.007	<0.001	<0.001	0

**Table A2:** Firth’s logistic regression to identify predictors of fatal outcomes.

Variable	Coefficient	Odds Ratio	95% CI Lower	95% CI Upper
Type of TB	−0.01	0.99	0.76	1.28
Age	−0.55	0.58	0.44	0.75
BMI	−0.06	0.94	0.73	1.23
SpO_2_ at diagnosis	0.21	1.23	0.95	1.6
Lowest SpO_2_	−0.56	0.57	0.44	0.74
CRP	−0.10	0.90	0.69	1.17
AST	−0.22	0.80	0.61	1.04
ALT	−0.07	0.93	0.71	1.21
LDH	0.39	1.47	1.13	1.91
IL6	0.51	1.67	1.29	2.17
D-dimer	0.002	1	0.77	1.3
Neutrophil count	0.26	1.3	1	1.68
Lymphocyte count	−0.76	0.47	0.36	0.61
Thrombocyte count	−0.20	0.82	0.63	1.06
CT involvement score	1.88	6.57	5.06	8.54
NLR	−0.23	0.79	0.61	1.03
PLR	0.15	1.16	0.89	1.51
SII	−0.11	0.9	0.69	1.17
